# Towards a Better Primary Healthcare in Europe: Shifts in Public Health Nutrition Policies

**DOI:** 10.3390/nu12113308

**Published:** 2020-10-29

**Authors:** Demosthenes B. Panagiotakos, Matina Kouvari, Kyriakos Souliotis

**Affiliations:** 1 Department of Nutrition and Dietetics, School of Health Science and Education, Harokopio University, 17671 Athens, Greece; matinakouvari4@gmail.com; 2 Faculty of Health, University of Canberra, Bruce ACT 2617, Australia; 3 Faculty of Social and Political Sciences, University of Peloponnese, 20100 Korinthos, Greece; ksouliotis@uop.gr

**Keywords:** health policies, nutrition policies, Europe, primary care

The interrelated challenges of suboptimal dietary habits and abnormal weight status have never been as high on the global and European public health agenda as nowadays [1]. Non-communicable diseases (NCDs), including cardiovascular diseases (CVD), cancer, diabetes and respiratory disease, kill 41 million people each year, equivalent to 71% of all deaths and >80% of premature deaths globally [2]. Obesity prevalence is either rapidly increasing or stabilizing at very high levels in almost all European countries [3]. On the other side, the latest results from the ongoing Global Burden of Disease Study revealed that one in five deaths globally can be attributed to an unhealthy diet, with this proportion soaring when abnormal weight status and other measures of maternal and child malnutrition are included [4]. Similarly, it is estimated that among all behaviors, nutrition makes the largest contribution to CVD mortality and morbidity at the population level across Europe [5].

Countries in the World Health Organization (WHO) European Region are rather diverse in terms of income and development levels, as well as food culture and traditions. However, their dietary habits are commonly suboptimal, characterized by energy imbalance and excessive intake of saturated fats, trans fatty acids, added sugars and salt—largely due to increased consumption of highly processed, energy-dense manufactured foods and sugar-sweetened beverages—as well as inadequate consumption of vegetables, fruits and whole grains [6]. Regions of low socioeconomic status are the most severely affected, with major economic and welfare costs for the whole society [7]. Such observations spur the need for ambitious action by European governments.

Comprehensive policies to promote healthy diets and prevent obesity in the European Region have been advocated by the WHO Regional Office for Europe since the adaptation of the first action plan in 2000 [8] (Figure 1). This action plan explicitly called on member states to introduce strategies on food and nutrition so as to reach the European Health21 target regarding healthier living, i.e., “By the year 2015, people across society should have adopted healthier patterns of living” [8]. This first action plan principally focused on the prevention of foodborne diseases as well as nutrition-related socioeconomic inequalities and food insecurity [8]. Since then, one third of the member states in the WHO European Region developed policies on food and nutrition and almost all had government-approved documents dealing with nutrition and food safety [9]. In 2008, a renewed nutrition action plan was launched [10]. This time, the action plan addressed the main public health challenges in the areas of nutrition, food safety and food security, dealing with diet-related NCDs (principally obesity), micronutrient deficiencies and foodborne diseases. A couple of years later, a number of “best buys” including recommended actions on salt and trans-fat consumption or limiting children’s exposure to advertising for foods high in saturated fats, sugars and salt were recognized [11]. In 2011, the Regional Committee adopted resolution EUR/RC61/R3 which endorsed the Action Plan for implementation of the European Strategy for the Prevention and Control of Non-communicable Diseases (NCD) 2012–2016 [12]. Three out of five priority interventions were related with “promotion of healthy consumption via fiscal and marketing policies”, “elimination of trans fats in food (and their replacement with polyunsaturated fats)” and “salt reduction”. In 2013, ministers of countries of the European Region adopted the “Vienna Declaration on Nutrition and Noncommunicable Diseases in the Context of Health 2020”. This declaration acknowledged that strategies to improve dietary health require government-led action in a broad range of areas and should be informed by increasing evidence of the efficacy of a comprehensive response incorporating a core set of policies. It also recognized that successful adoption and implementation of these policies requires continuing emphasis on health-in-all-policies and whole-of-government approaches for the creation of healthy and sustainable food systems, in line with the European Health 2020 strategy [13]. Following this, the global action plan for the prevention and control of NCDs was launched, setting a 25% relative reduction in overall mortality of cardiovascular disease, diabetes, cancer and respiratory diseases as well as describing specific nutrition-related targets [14]. To meet this challenge, the European Food and Nutrition Action Plan 2015–2020 was endorsed by member states. This action plan included state-of-the-art knowledge on the factors that influence dietary behavior throughout the life-course and policies and interventions for a wide range of settings and domains [15].

Within the most recent action plan, member states had to develop common tools, share experiences, improve the availability of data and enhance capacity for monitoring and surveillance so as to halt increases in obesity and diabetes, halt the increase in the prevalence of overweight status among children under five years old, reduce the mean population intake of salt and sodium by 30%, increase the rate of exclusive breastfeeding in the first six months of life to at least 50% and reduce the proportion of stunted children under five years by 40% as well as the prevalence of anemia among non-pregnant women of reproductive age by 50%. All of these have been set as the global nutrition targets for 2025 [16]. Policy options that governments might consider included the creation of healthy food environments from school to food markets. In particular, labelling trans fatty acids content and food and beverage reformulation—to tackle nutrient deficiencies—as well as setting specific regulations regarding the marketing of food products, especially toward children, have been developed. Additionally, the promotion of healthy dietary habits during pregnancy as well as early in life was highlighted. Moreover, this action plan underlined the role of health professionals in offering nutrition counselling, particularly in the primary health care context and the need for public to be provided with nutritional skills and capacity. Finally, surveillance, monitoring and evaluation of the applied policies, including monitoring the growth of children under five years old or assessment of individuals’ dietary habits through representative national surveys, were indispensable parts of this action plan [15].

According to the latest report of the European Commission, more than 750,000 deaths per year are attributed to behavioral factors, with nutrition and increased weight status being on the short list [17]. The role of food environments in positively or negatively affecting people’s food choices, dietary behavior and, subsequently, health outcomes has been well described in the literature. Nowadays, a suite of policies are recognized as essential for creating a healthy food environment on a national and European basis. The importance of nutrition throughout life-course has been well understood and appreciated to prevent obesity and NCDs. Towards this need, tailor-made policies are demanded to effectively target each different life stage. On the other side, the health system demonstrates a major role in promoting healthy dietary behaviors. In this context, practice and training for health professionals might have to be transformed, including investment in more diversified human resources at primary care level.

All of these are put under the umbrella of primary care strategies. Considering that in the meanwhile, only 3% of total health expenditures in EU Member States is devoted to primary care, many things remain to be done. Besides the fact that significant progress has been made in various areas of public health nutrition, the European Region is not fully on-track to achieve the global NCD targets. Therefore, more ambitious and comprehensive nutrition policies should be prioritized at a faster pace, accompanied by a more robust monitoring system to discriminate progress and to guide timely and effective policies.

## Figures and Tables

**Figure 1:**
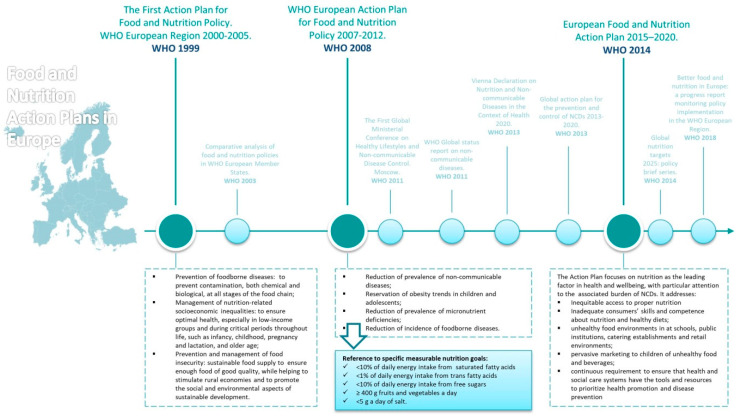
Public health and nutrition policies in Europe. Abbreviations: NCD (non-communicable diseases); WHO (World Health Organization). Source of information included in figure: [8,9,10,11,12,13,14,15].
